# Assessment of Two Advanced Aluminium-Based Metal Matrix Composites for Application to High Energy Physics Detectors

**DOI:** 10.3390/ma16010268

**Published:** 2022-12-27

**Authors:** Katie Elizabeth Buchanan, Stefano Sgobba, Michal Dalemir Celuch, Francisco Perez Gomez, Antti Onnela, Pierre Rose, Hans Postema, Mariano Pentella, Guillaume Lacombe, Benjamin Thomas, Renaud de Langlade, Yvan Paquin

**Affiliations:** 1 European Organization for Nuclear Research (CERN), 1211 Geneva, Switzerland; 2 Department of Physics, Cornell University, Ithaca, NY 14850, USA; 3 MINAPACK, 38510 Vezeronce Curtin, France; 4 NOVAPACK Technologies, 38510 Vezeronce Curtin, France

**Keywords:** Al/C_f_ metal matrix composites, CERN compact muon solenoid (CMS), liquid casting, semi-liquid phase sintering, galvanic corrosion, powder metallurgy

## Abstract

The Outer Tracker of the Compact Muon Solenoid (CMS), one of the large experiments at the CERN Large Hadron Collider, will consist of about 13,200 modules, each built up of two silicon sensors. The modules and support structures include thousands of parts that contribute to positioning and cooling the sensors during operation at −30 °C. These parts should be low mass while featuring high thermal conductivity, stiffness and strength. Their thermal expansion coefficient should match that of silicon to avoid deformations during cooling cycles. Due to their unique thermal and mechanical properties, aluminium-carbon fibre (Al/C_f_) Metal Matrix Composites are the material of choice to produce such light and stable thermal management components for High Energy Physics detectors. For the CMS Outer Tracker, about 500,000 cm^3^ of Al/C_f_ raw material will be required to be produced through a reliable process to guarantee consistent properties throughout parts manufacturing. Two Al/C_f_ production routes are currently considered: liquid casting by gas-pressure infiltration and a powder metallurgy process based on continuous semi-liquid phase sintering. The dimensional stability of the resulting material is of paramount importance. Irreversible change of shape may be induced by moisture adsorption and the onset of galvanic corrosion at the discontinuous interfaces between C_f_ and Al. This paper presents the results of an extensive investigation through Computed Microtomography, direct microscopical investigations, analysis of the interfaces and metrology measurements aimed at comparing and interpreting the response to different environments of the respective products. The results obtained confirm the suitability of the two investigated Al/Cf MMCs for application to components of the CMS Outer Tracker, requiring tight geometrical control and microstructural stability over time. However, for PM parts sintered through the semi-liquid phase process, a multilayered protective noble metal coating is necessary the make them impervious to moisture, allowing dimensional stability to be guaranteed and the onset of corrosion phenomena to be avoided, while the product obtained by gas-pressure infiltration has shown less sensitive even to extreme temperature-humidity cycles and may be used uncoated.

## 1. Introduction

**Figure 1:**
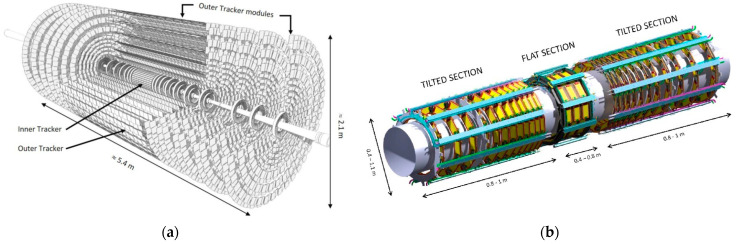
(**a**) Perspective view of the new Outer and Inner Trackers for CMS. The small rectangles that populate the disks at the ends and the concentric cylinders at the centre of the structure are the modules of the tracker. The cylinders are referred to as the barrel part of the tracker. (**b**) The innermost layer of the Outer Tracker barrel section is subdivided into a central section (flat) and two tilted sections at both ends (from [4]). The yellow rectangles in the figures are the silicon sensor modules, horizontal (tilted) in the central (end) sections. The central part of the Inner Tracker support tube is also visible inside.

The Compact Muon Solenoid (CMS) is a general-purpose detector at the Large Hadron Collider (LHC) at CERN, aimed at studying particles produced in high-energy proton-proton and heavy ion collisions [1]. The current LHC programme will end in 2025 with Run 3. Afterwards, the LHC will be upgraded to operate at significantly higher luminosity in the framework of the High Luminosity Large Hadron Collider (HL-LHC) project, which will achieve instantaneous luminosities a factor of five larger than the LHC nominal value [2]. The upgraded machine will begin operation in 2029. The experiments will enlarge their data sampling by one order of magnitude compared with the current LHC programme [3]. In particular, more particles will impinge on the CMS detector than its detector systems were designed to handle. Therefore, an extensive upgrade of the CMS detector will take place in the coming years. One of the detector systems which will be entirely replaced to fully exploit the delivered luminosity is the tracker, which measures the trajectories of charged particles from the proton-proton collisions. The current strip and pixel trackers will be replaced by new Outer and Inner Tracker detectors. Their layout and innermost layers are shown in Figure 1a. Figure 1b shows a sub-section of the new tracker, the TBPS (Tracker Barrel with Pixel-Strip modules), of which prototypes have been manufactured.

The Outer Tracker will consist of about 13,200 modules, each built up of two silicon sensors separated by mechanical spacers [5]. The modules and support structures include thousands of parts that contribute to positioning and cooling the sensors during operation at −30 °C. These parts need low mass while featuring high thermal conductivity, stiffness and strength. Their thermal expansion coefficient (CTE) should match as closely as possible that of silicon 2.4 × 10^−6^ K^−1^ to avoid deformations during cooling cycles. In particular, CTE should be at most 4 × 10^−6^ K^−1^ in the part planes parallel to silicon sensors and 2.4 × 10^−5^ K^−1^ in the through-thickness direction. The thermal conductivity of the mechanical module parts is specified to be at least 120 W/mK and above 220 W/mK in the most critical areas. A Young’s modulus > 95 GPa and flexural strength of min. 120 MPa are required. Relative density should be at most 2600 kg/m^3^ and homogeneous throughout the volume of the parts. Moreover, the parts should be completely non-magnetic (relative magnetic permeability *µ_r_* ≤ 1.005) at the operating field of the superconducting CMS solenoid magnet of 3.5 T.

The modules have to feature dimensional and metallurgical stability during exposure to unregulated temperature and moisture conditions. Indeed, during storage and handling, as well as following installation in the caverns of the experiments, they may be submitted to uncontrolled temperature and moisture conditions before the steady operation at −30 °C.

Since the early 90 s, Metal Matrix Composites (MMCs) have been looked at as reference material for thermal management solutions for electronic packaging. Their properties, in particular CTE, can be tailored to match one of semiconductors in particular Si, by a proper selection of the reinforcement, its orientation and volume fraction. For High Energy Physics (HEP) detectors, the selected materials have to be as transparent as possible to particles. A “material budget” equivalent in terms of thickness to less than 1% of the radiation length of the material is considered a common requirement [6]. For this reason, aluminium or aluminium alloy matrix are an advantage against higher-density copper matrix composites. Although aluminium has much lower conductivity than pure copper, in aluminium-carbon fibre (Al/C_f_) composites, the thermal conductivity values specified for the CMS Outer Tracker application can be obtained. For a 58 vol.% unidirectional C_f_ content in an Al-3 wt.% Mg matrix MMC produced by gas pressure infiltration, the thermal conductivity of 540 W/mK could be achieved [7], while 273 W/mK in the plane were reported that for a unidirectional C_f_ product obtained by a low-pressure infiltration process [8]. Even for a short C_f_ reinforcement, Silvain et al. [9] reported for a sintered MMC based on an Al + 5 vol.% AlSi matrix and 40 vol.% carbon fibres, thermal conductivity as high as 240 W/mK in the plane and the order of 120 W/mK in the transverse direction of carbon fibres. Ceteris paribus, these values were much higher than measured by the same authors for an Al/C_f_ product (in the order of 120 W/mK and 65 W/mK, respectively).

**Figure 2:**
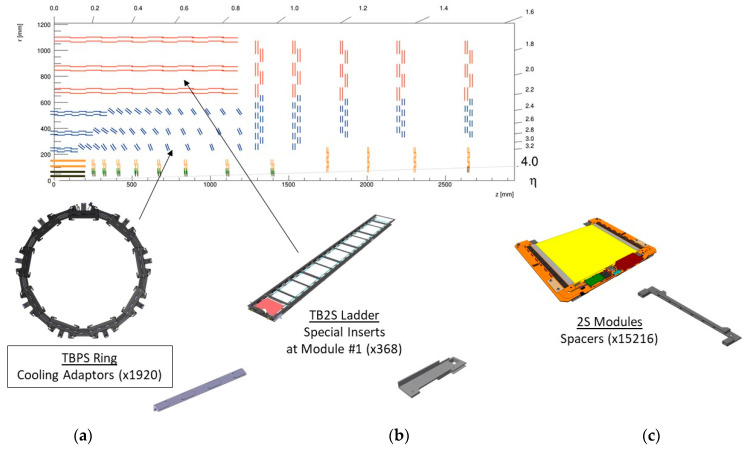
Al/C_f_ components inside the CMS Outer Tracker. Al/C_f_ is applied to produce parts for Rings (**a**), Ladders (**b**) and Modules (**c**). Parts made of Al/C_f_ act as supports and thermal interfaces for the modules. The Al/C_f_ parts needed for the Rings are triangular in shape; hence they have to be 3D machined. There are seven types, each with a specific angle.

Due to the above properties, Al/C_f_ Metal Matrix Composites are the material of choice to produce light and stable thermal management components for High Energy Physics detectors. In particular, for the CMS Outer Tracker, Al/C_f_ is retained against a ceramic material solution such as AlN. Indeed, the CTE of the latter is in the order of 4 × 10^−6^ K^−1^ to 5 × 10^−6^ K^−1^ with little dependence on the directions, hence an advantage against Al/C_f_ MMCs, which are highly anisotropic. However, AlN, which is selected for some parts of ATLAS and CMS detectors, can only be used for components requiring to be electrical insulators. Moreover, AlN is not easily machinable in three-dimensional shapes as it is required, for example, for the cooling adapters of the Outer Tracker: AlN parts can only be obtained by water jet or laser cutting in two-dimensional shapes, hence only suitable and used for flat components of the detectors such as spacers, carrier plates etc. Finally, for the Outer Tracker, the parts need to be electrically conductive, which is the case for Al/C_f_ but not for AlN. Based on the above, about 500,000 cm^3^ of Al/C_f_ raw material will be ordered for the build of different parts of the CMS Outer Tracker (Figure 2), to be produced through a reliable process to guarantee consistent properties all along part manufacturing. In particular, it is critical for the function of the modules that the machined parts achieve tight dimensional tolerances and that their dimensional stability is guaranteed over time to a few microns.

One of the drawbacks of MMCs with aluminium alloy matrix, and in particular of Al/C_f_, is their sensitivity to corrosion, even in normal humidity air [10]. Indeed, inhomogeneities are introduced on the surface of the parts exposed to the environment, featuring a combination of galvanically active metal and reinforcement material, such as graphite or C_f_, which is more noble in the galvanic potential series [11,12,13]. Severe galvanic corrosion phenomena induce dimensional changes and the formation of internal defects near corrosion surfaces and in the bulk of the parts. To circumvent this issue, the applicability of a noble metal coating consisting of nickel-based layers covered by a thin gold film is studied in the present paper. It is known that noble metal coatings may suffer from corrosion at flaw sites in case of imperfect covering or local discontinuities in the film due to unfavourable anodic (aluminium) to cathodic (noble coating) coupling and area ratio in the presence of a local defect [11]. For this reason, their efficiency as barrier protection is addressed in the present paper, together with their magnetic properties, the latter being particularly relevant for the specific case of HEP applications.

## 2. Materials and Methods

Two different Al/C_f_ products are the object of the present investigation, both reinforced by chopped high elastic modulus carbon fibres oriented in the xy plane. The volume fraction of carbon fibres is controlled to target an in-plane CTE of 4 × 10^−6^ K^−1^ and is in the order of 50 vol.%. They are both easily machinable to tight tolerances.

The first product, Al MetGraf™ 4-230 (hereafter referred to as GPI) manufactured by Parker Hannifin Corporation (Parker Hannifin Engineered Materials Group, North Haven CT USA), is obtained by gas-pressure assisted metal infiltration casting [14]. It is based on an AlSi alloy matrix (high purity AlSi eutectic alloy A413 HP) reinforced with high modulus pitch-based carbon fibres. The gas-pressure infiltration process is based on a preform infiltrated by the molten A413 HP alloy. The product incorporates chopped fibres randomly oriented in a felted preform [15], whose content (proprietary information) is aimed at achieving a density of 2400 kg/m^3^ and an in-plane (through-thickness) average CTE of 4 × 10^−6^ K^−1^ (24 × 10^−6^ K^−1^ ) in the temperature range −55 °C to +125 °C. According to the manufacturer’s data, it features an in-plane (through-thickness) thermal conductivity of 230 (120) W/mK, Young’s modulus of 98 GPa, a flexural strength of 186 MPa and an ultimate in-plane tensile strength of 103 MPa, compliant with the Outer Tracker specification limits.

The second product, which was manufactured by the company Composite Innovation (hereafter referred to as PM), is a powder metallurgy MMC based on a matrix obtained from a mixture of spherical pure Al powder and 5 vol.% of eutectic Al11.3at%Si powder, engineered by the Institute of Condensed Matter Chemistry (ICMCB) of the Centre National de la Recherche Scientifique (CNRS) of Bordeaux (France). It is sintered in a semi-liquid process. The mixed powder was hot pressed by applying a 60 MPa uniaxial pressure for a holding time of 30 min at 600 °C, between the melting points of the Al11.3%Si alloy (585 °C) and of pure Al (660 °C), respectively. Details of the constituents and the process are provided in [16].

**Figure 3:**
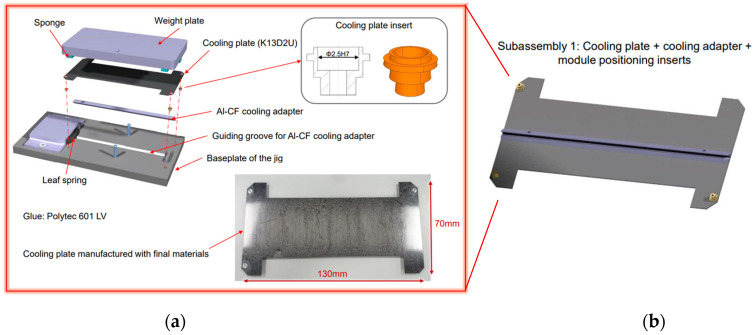
Supplied parts for investigation include subassemblies for the TBPS. (**a**) The details of the subassembly procedure are illustrated. The subassemblies consist of an Al/C_f_ cooling adapter glued on a carbon-fibre/cyanate ester composite cooling plate with aluminium inserts for module positioning. (**b**) Standalone cooling adapters have also been supplied, with and without coating.

Cooling adapters fully machined from Al/C_f_ blocks of each of the two products were studied, as well as simpler rectangular cross-section samples cut from the blocks. In some cases, the adapters were glued onto the carbon plates using a Polytec EP 601-LV glue and cured for 48 h at RT (25 °C) with ≈50% RH (Figure 3). The carbon plates consist of five plies of 120 gsm K13D2U/EX-1515 UD carbon fibre/Toray cyanate ester prepreg resin system, laminated to ~0.5 mm thickness and featuring a [0, 90, 0, 90, 0] layup.

Volumetric examinations of the parts were performed by X-ray micro-tomography (CT) at CERN with the help of a Zeiss METROTOM 1500 tomograph (Carl Zeiss, Oberkochen, Germany). The achieved resolution (voxel size) is in the order of 15 µm to 20 µm. The same device was used for 3D metrological inspections with the same resolution.

Optical inspections at reception were performed using a Keyence VR-3200-G2 microscope. Metallurgical observations were carried out with the help of a Zeiss AxioImager microscope and a Keyence VHX-6000 Digital Microscope (DM, Keyence Corporation, Osaka, Japan). SEM and FIB-SEM observations were performed respectively with Zeiss Sigma and Sigma 500 Field Emission Gun (FEG, Carl Zeiss, Oberkochen, Germany) systems and a Zeiss XB540 FIB-SEM unit. Secondary Electrons (SE), Backscattered Electrons (BSE) modes and Energy dispersive X-ray Spectroscopy (EDS) were applied in order to identify and analyse the distribution of the phases in the bulk of the material, as well as the corrosion residues and their nature.

Magnetic permeability measurements were performed with the help of a portable Foerster Magnetoscop 1.069^®^ device (Institut Dr. Foerster GmbH & Co., Reutlingen, Germany) [17] under a constant field of 80 kA/m (about 0.1 T). The Foerster Magnetoscop is a commercial instrument used to measure the relative permeability of feebly magnetic specimens. This flux-distortion detection method is compliant with Test Method 4 of ASTM A342/A342M [18]. The instrument is equipped with a permeability probe made of a cylindrical permanent magnet and two fluxgate magnetometers. The specimens were also tested by a Static-Sample Magnetometer (SSM) available at CERN in order to verify the permeability values under a wider applied field range (up to 1 T) and benchmark the Foerster Magnetoscop 1.069^®^ results at 0.1 T for future inspections of the adapters following coating process to be eventually applied for the series production. SSM measurements consist of the insertion of the specimen to be tested inside a dipolar and highly uniform magnetic field and a measurement of the perturbation of such field induced by the specimen, from which the relative magnetic permeability of the specimen is calculated. A description of the technique is available in [19].

## 3. Results

### 3.1. Non-Destructive Examinations and Dimensional Metrology

Prototype subassemblies illustrated in Figure 3 were manufactured using PM cooling adapters glued to the carbon fibre plate. The flatness of the cooling plate, as measured before the assembly using the Keyence VR-3200-G2 microscope, was 120 µm, while the flatness was up to 200 µm after gluing and curing. These prototypes were then stored in an unregulated environment, in particular humidity, with temperatures ranging from 18 °C to 35 °C for three months. Observations taken after this storage time showed a significant increase in the deflection up to 400 µm.

The homogeneity and the soundness of the internal structure of additional subassemblies were also examined through CT scans. CT also allowed their dimensions to be precisely assessed against specified dimensions. Four samples, with adapters milled to final shape by MINAPACK, were compared, three including a PM and one a GPI cooling adapter for comparison, namely:

- No.1—Subassembly with a PM adapter-Reference sample stored in temperature and humidity-controlled laboratory conditions
- No.10—Subassembly with a PM adapter-Sample stored in unregulated conditions of temperature and humidity for three months (see above)
- No.25—Subassembly with a PM adapter-Sample stored in extreme controlled conditions of temperature (70 °C) and relative humidity (RH = 100%) for 60 h
- No.7—Subassembly with a GPI adapter stored in extreme controlled conditions of temperature and humidity (as above for sample No.25)

**Figure 4:**
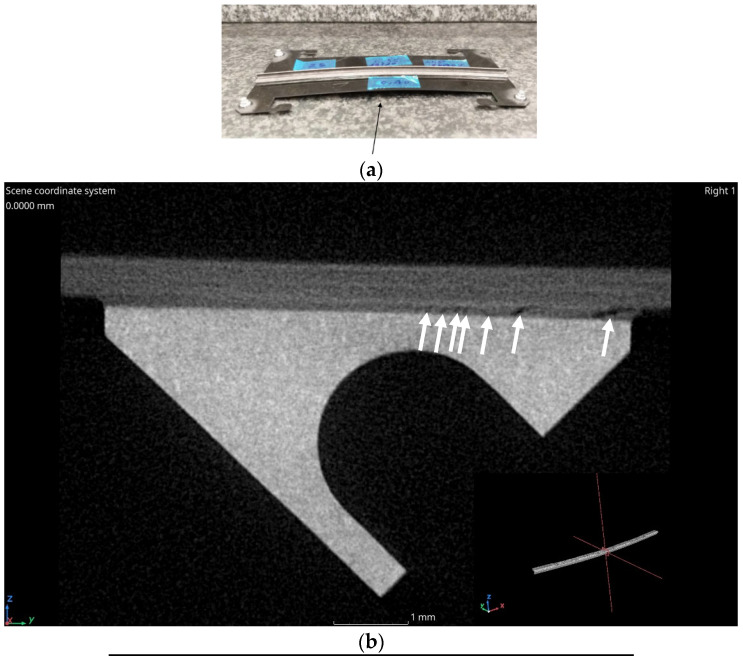

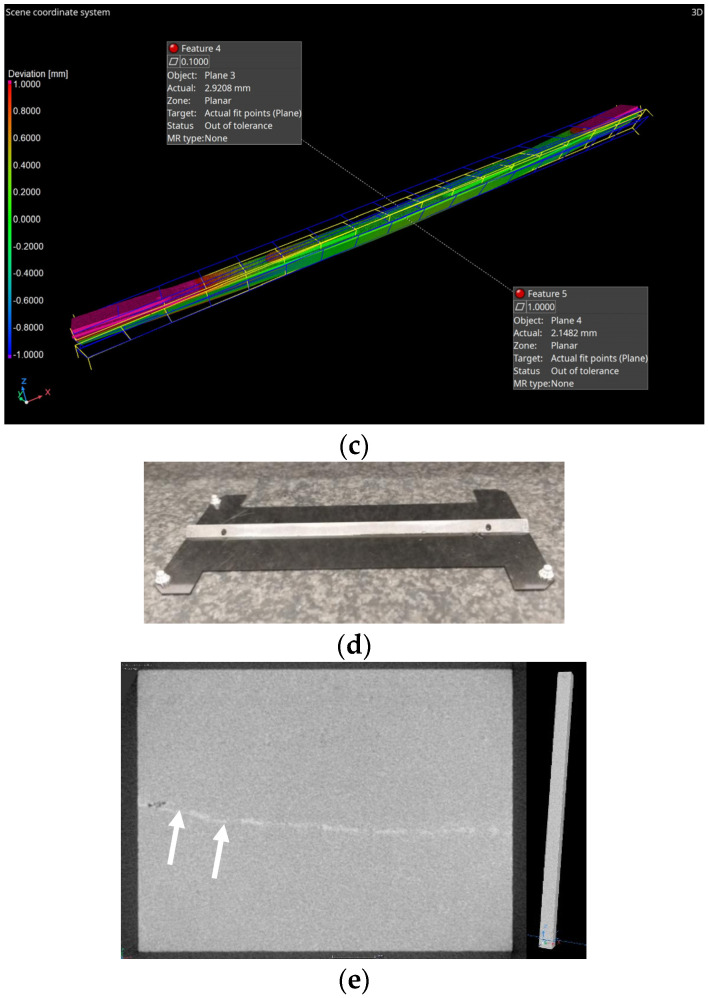
(**a**) General view showing severe bending and (**b**) CT cross-section of sample No.25 stored in extreme conditions. Flaws (identified by arrows) emerge in the glue volume. (**c**) The out-of-flatness is 2.9 mm while the lateral deflection is 2.15 mm. (**d**) General view of a subassembly including a GPI adapter. (**e**) CT cross-sectional view of the GPI adapter (sample No.7), also exposed to extreme conditions. Segregation bands and cavities (identified by arrows) are present in the bulk of the adapter, but there is no deformation of the sample.

The reference sample (No.1) did not show any degradation of the geometry and remained within 70 µm of flatness. CT detected only some small voids in the adapter. Due to the exposure, sample No.10 developed a bending in the order of 300 µm; moreover, flaws are present in the glued interface. Sample No.25 developed the largest bending (2.9 mm) and the highest density of flaws in the glue volume, while sample No.7 (GPI adapter) shows casting imperfections (a centreline segregation band, typically observed in AlSi-based castings and pores that, as expected tend to be enclosed by the segregation band [20]) but remained substantially flat despite the same extreme conditions of storage applied to PM sample No.25 (Figure 4).

**Figure 5:**
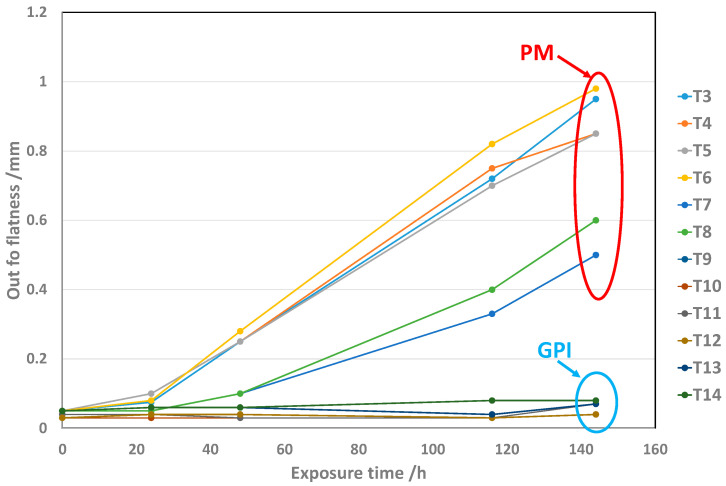
Evolution of the deformation in the function of time of subassemblies, including PM or GPI adapters, respectively. The subassemblies were exposed to 50 °C and 100% RH for increasing times up to 144 h. Their deformation was regularly measured during the exposure. The deformation reported in the y-axis represents the maximum measured sagitta for the respective times of exposure.

Conventional metrology measurements performed on a large number of subassemblies, including PM and GPI adapters, respectively, confirmed that the former underwent a high deformation following the temperature-humidity cycles, while the latter remained substantially undeformed under the same conditions (Figure 5).

### 3.2. Destructive Examinations

**Figure 6:**
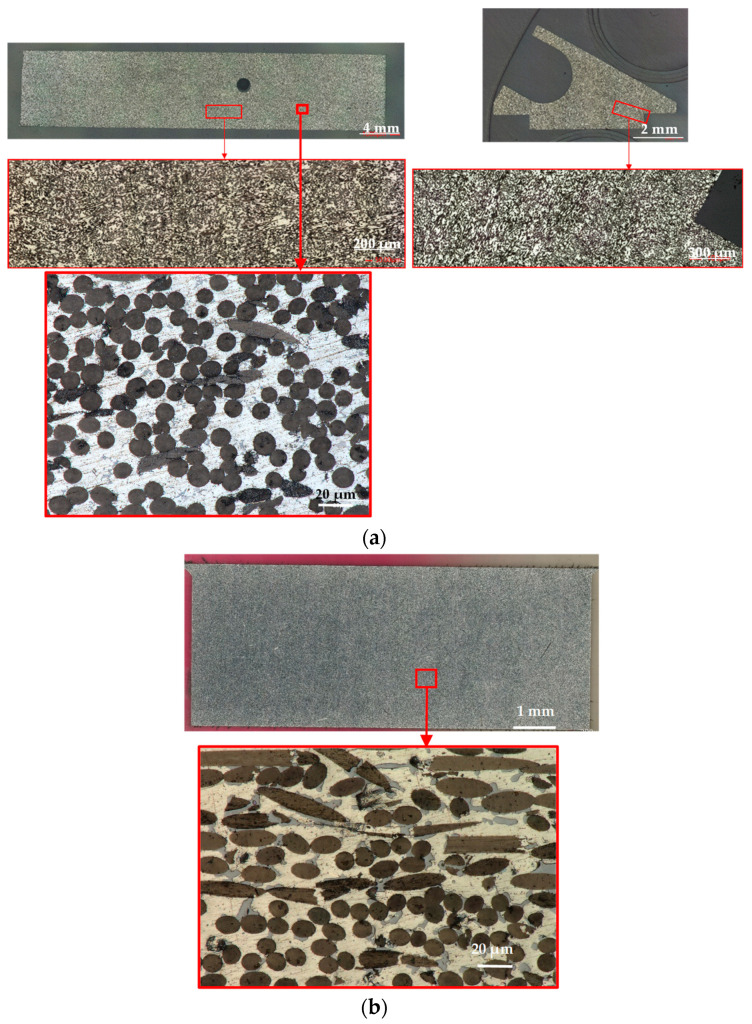
(**a**) Example of longitudinal (left) and transverse (right) metallographic cut of a PM adapter (reference sample, stored in laboratory conditions). The microstructure consists of randomly oriented C_f_ (dark grey) in a matrix of Al (light grey) and eutectic AlSi (medium grey) phases; see also Figure 7. (**b**) Longitudinal metallographic cross-cut of a GPI adapter (as above). The microstructure consists of randomly oriented C_f_ (dark grey) surrounded by the eutectic matrix featuring Al-rich (yellow) and Si-rich phases (light grey); see also Figure 10.

**Figure 7:**
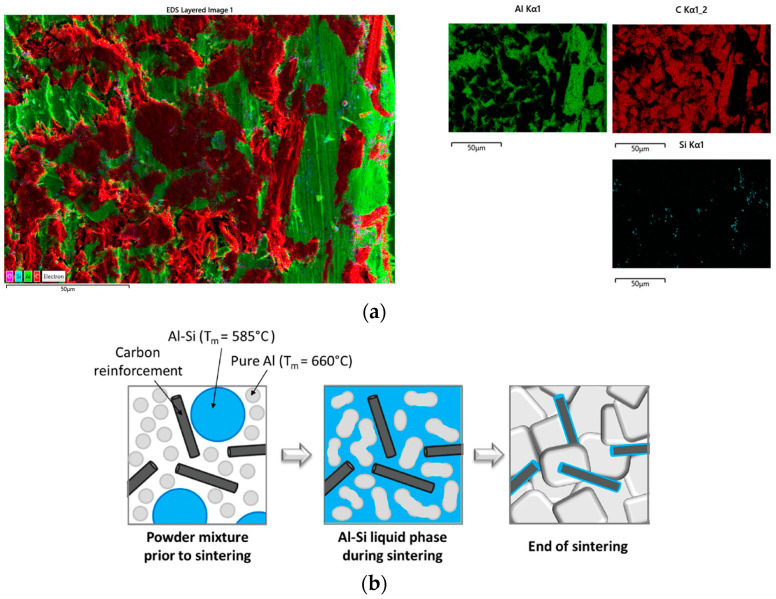
(**a**) EDS mapping was performed on a cross-section of a reference PM adapter, showing the distribution of Al, C and Si. (**b**) A Si-rich phase is expected due to the addition of eutectic Al11.3%Si powder in the sintering process (from [16]).

Transverse and longitudinal metallographic cuts were performed to assess the distribution and orientation of the carbon fibres in both PM and GPI adapters. Digital microscopy images showed homogeneous distribution of fibres in the PM samples, with orientation mostly longitudinal, as observable in the transverse crosscut. This confirms the prevailing in-plane orientation of the fibres. However, groups of misoriented fibres were also widely observed (Figure 6a). GPI adapters feature a similar fibre distribution (Figure 6b). Additional SEM observations and EDS analyses were performed on as-received PM samples, showing that the C_f_s is surrounded by the Al matrix and locally by the AlSi eutectic (Si is detected by EDS in association with Al in the vicinity of the fibres). The local presence of Si as an alloying element is expected in association with the applied semi-liquid phase sintering process (Figure 7).

**Figure 8:**
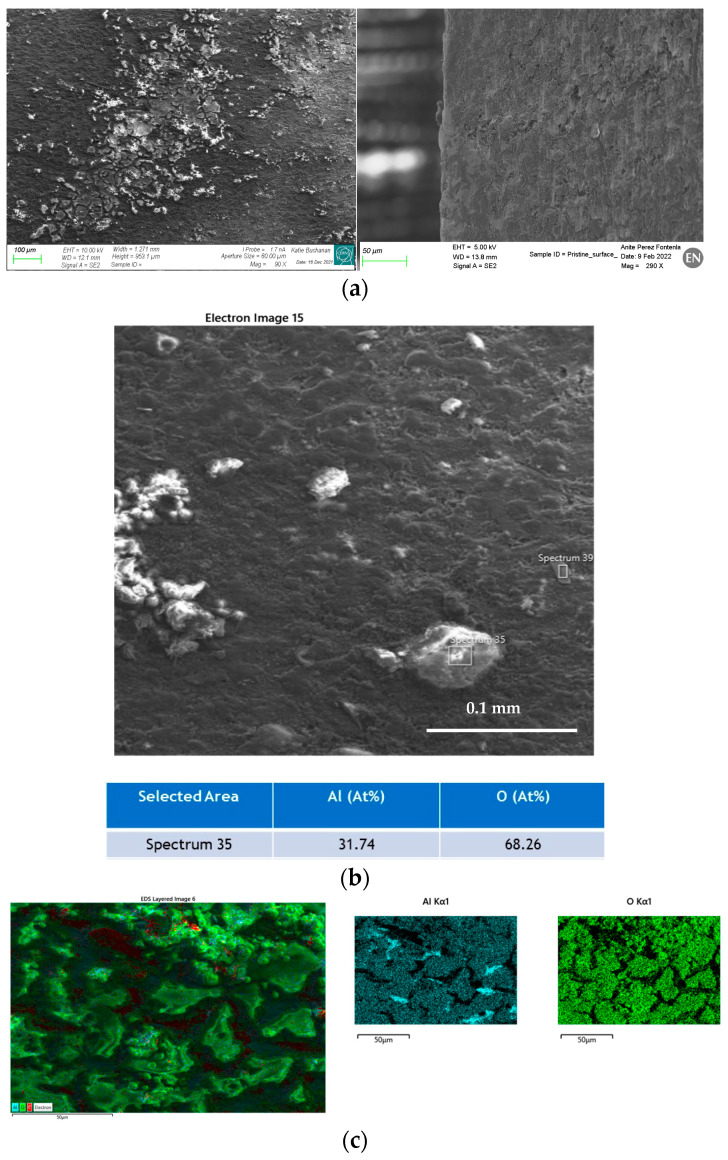
(**a**) Left: SEM view of the outer surface of the PM sample subjected to a temperature (70 °C) and humidity (RH = 100%) cycle for 60 h. Evidence of corrosion spots and corrosion residues (whitish phase) featuring poor adherence on the surface. Right: as above, the GPI sample features large areas with no visible corrosion events. (**b**) EDS analysis of the whitish phase of the PM sample, mainly consisting of Al and O (32 at.% Al and 68 at.% O), close to Al_2_O_3_ composition. (**c**) EDS mapping of the distribution of Al and O on the sample surface.

The outer surfaces of a PM and GPI sample, respectively, exposed to the temperature-humidity cycle, were also examined by SEM prior to eventual cross-cuts in order to assess the influence of the cycle on the onset of possible corrosion phenomena. Severe corrosion spots and corrosion residues were observed on the surface of the PM sample (Figure 8), while on the GPI sample, only a little evidence of corrosion was identified on the sample surface.

**Figure 9:**
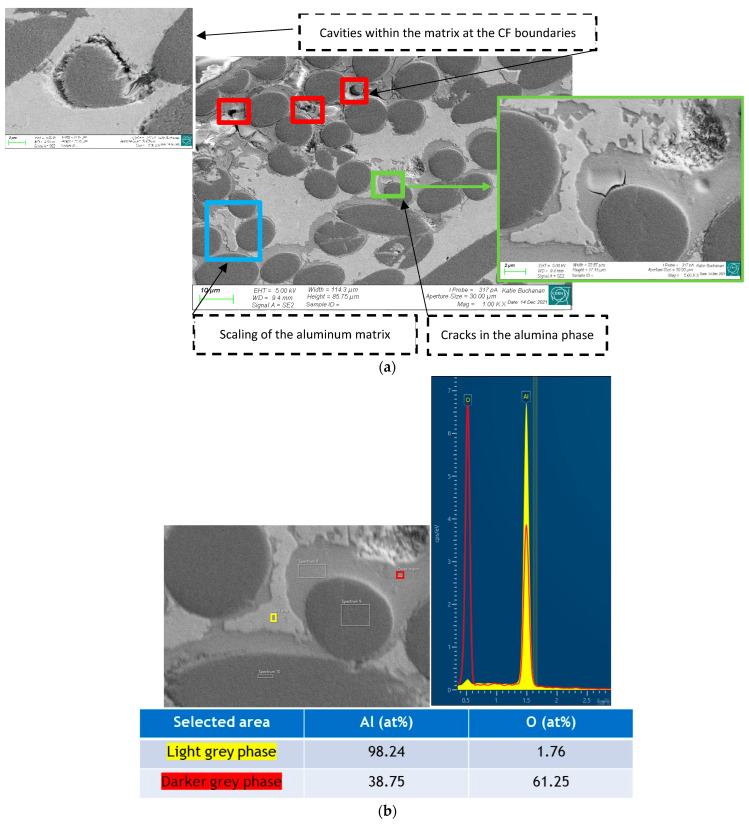
(**a**) Transverse cross-sectional cut of the PM sample shown in Figure 8. Cavities are present at the boundary between the Al and the C_f_s, locally filled with corrosion residues. The grey phase (identified by an arrow), whose stoichiometry is close to alumina, features a brittle appearance with cracks developing close to the C_f_s. (**b**) EDS analysis of the dark grey phase shows a composition close to Al_2_O_3_ (Al 38.75 at.% and O 61.25 at.%), while the adjacent matrix is almost pure Al (98.24 at.%).

Observations of sample cross-sections were carried out in order to assess if there was any internal damage in the parts. SEM examination of a PM sample exposed to a temperature (70 °C) and humidity (RH = 100%) cycle for 60 h (Figure 9) showed a darker grey phase surrounding the C_f_s that is present in a higher concentration towards the edges of the sample. The phase has a brittle appearance, with cracks originating close to the C_f_ boundary. EDS analysis of this phase (Figure 9b) shows a composition close to Al_2_O_3_ (Al 38.75 at.% and O 61.25 at.%), while the adjacent matrix is almost pure Al (98.24 at.%). Cavities are observed at the boundary between the Al and the C_f_ s. They are synonymous with an imperfect bonding of the fibres to the matrix. Moreover, in some positions, they are associated with corrosion residues due to the intake of moisture in case they have connectivity to the surface through the open channel of cavities along the fibres.

**Figure 10:**
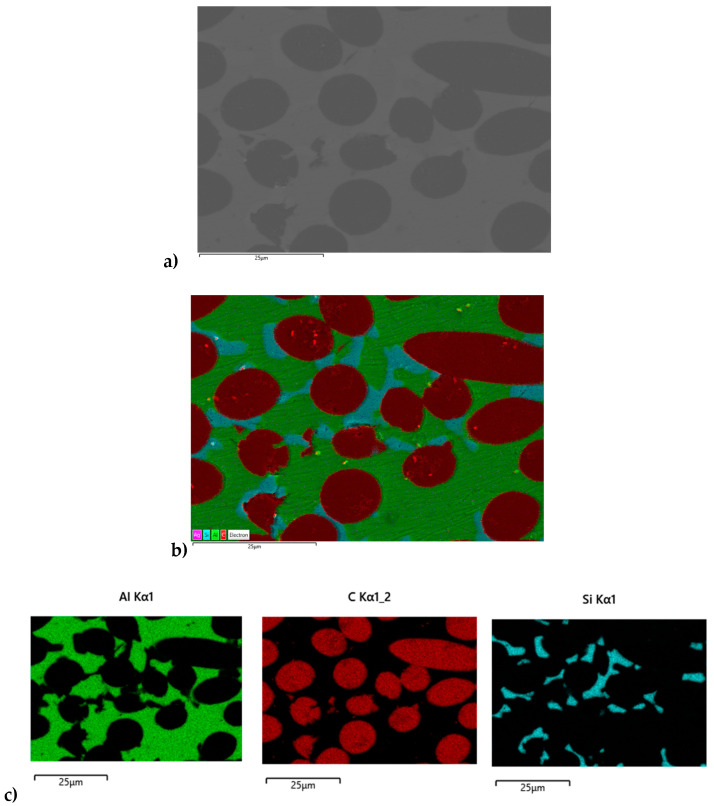
(**a**) SEM observation of a transverse cross-sectional cut of the GPI sample subjected to the same temperature and humidity cycle as the PM sample illustrated in Figure 8 and Figure 9. No signs of internal corrosion are apparent. A continuous interface bonding between the C_f_s and the matrix is observed. (**b**,**c**) EDS mapping of the phases showing, in addition to the C-fibres, an Al-rich and a Si-rich phase in the AlSi eutectic alloy matrix.

Figure 10 illustrates the microstructure of a GPI sample submitted to the same extreme cycle of temperature and humidity as the PM sample. No signs of internal corrosion are apparent, and a continuous interface bonding between the C_f_ and the matrix is observed.

From the above observations, it is confirmed that the PM samples feature an open structure with a network of cavities between the C_f_s and the matrix. They are preferential sites for the onset of galvanic corrosion: moisture pick-up induces active galvanic corrosion phenomena in the Al-based matrix, which is highly anodic to the C_f_. These PM products show extreme moisture sensitivity due to their open structure issued from the fabrication route. On the contrary, similar observations of GPI products confirm that they do not develop significant corrosion due to a more compact structure (Figure 10) and remain more dimensionally stable. Instead, CT scans and destructive observations of the parts confirm that the GPI sample contains unreinforced segregation bands, which are not present in the PM products and are mechanically weak. Indeed, developments of flaws in these bands were observed as a consequence of machining or assembly operations.

### 3.3. Examination of Coatings and Their Effect on Magnetic Properties

Based on the above results, in order to improve corrosion protection against heat and intake of moisture, the PM products have been subjected to surface treatment under the form of a noble metal coating. An electroless NiP coating of a nominal thickness of 12 µm was applied to the surface, followed by a sequence of electrolytic Ni and Au coatings, the latter of a nominal thickness of 0.2 µm. The coating process was according to MIL-STD-883L [21]. In the process, a baking cycle (1 h at 250 °C) was included for hydrogen outgassing. No blisters, pinholes, cracks or delamination were observed following the process.

**Figure 11:**
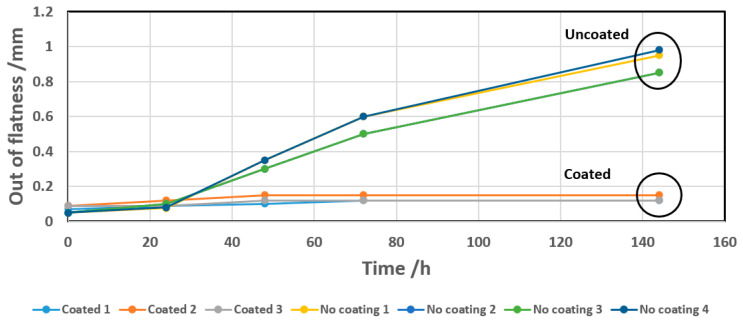
Evolution as a function of time of the deformation (out of flatness) of subassemblies, including coated and uncoated PM adapters, exposed to a temperature-humidity cycle (50 °C, 100% RH up to 144 h).

Subassemblies, including coated PM adapters (and uncoated for comparison) glued to C_f_ cooling plates, were then subjected to the same temperature-humidity cycle as depicted in Figure 5 (50 °C, 100% RH, exposure up to 144 h). The subassemblies, including coated adapters, have shown dimensional stability, while the ones with uncoated adapters develop out of flatness in the range of 0.8 mm to 1 mm at the end of the exposure (Figure 11).

**Figure 12:**
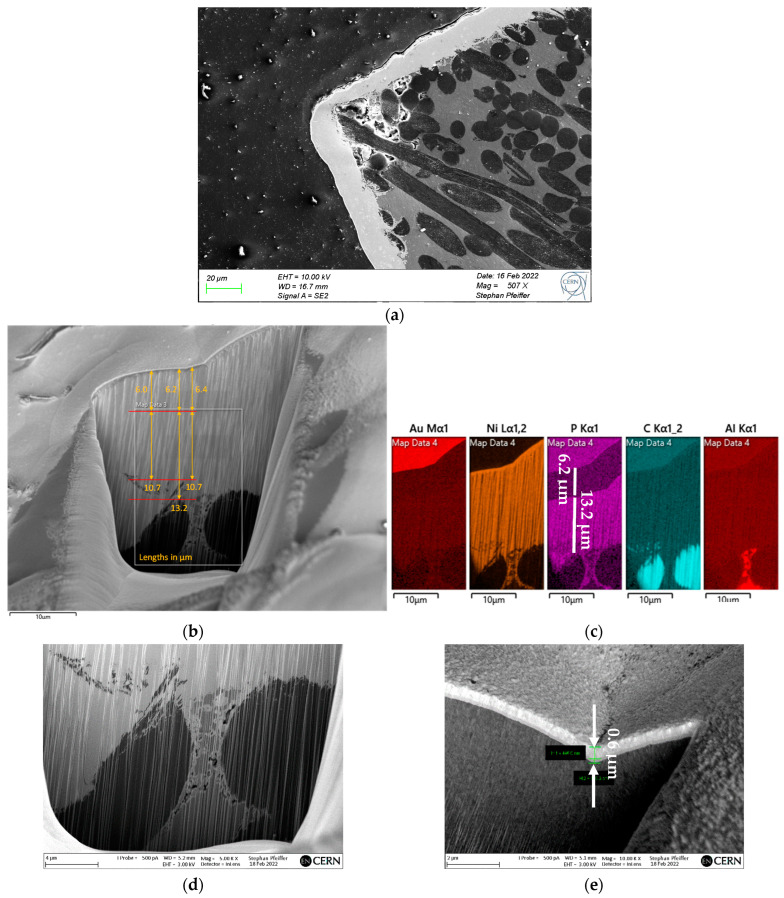

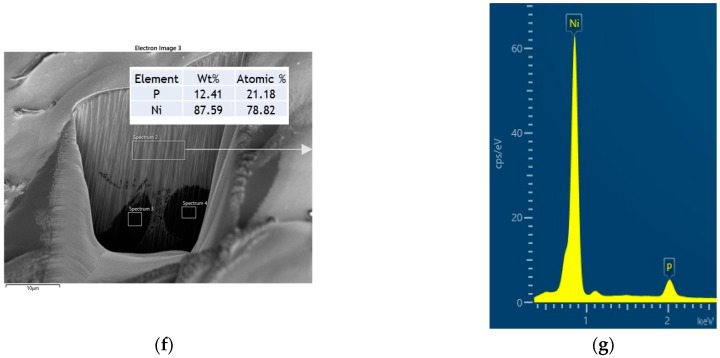
SEM and FIB-SEM examinations of the coating. (**a**) Cross-sectional cut of the coating observed by SEM, showing uniform thickness of the coating except for the edges, where the thickness is lower. However, no underlying corrosion events or discontinuities are observed in the coating impervious to moisture. (**b**) FIB-SEM observations allowed us to quantify the total thickness of the coating, in the order of 20 µm, and the thickness of the individual NiP and pure Ni constituent layers, respectively. (**c**) The EDS analysis in a selected area adjacent to the C_f_ shows the signal of both Ni and P for the layer closer to the substrate. Good wetting of the C_f_s by the coating is confirmed. (**d**) close view of the interface between the Al matrix and the C_f_s, confirming good wetting by the NiP deposit and limited unfilled cavities. (**e**) Assessment of the Au coating, showing a typical thickness of 0.6 µm (for a nominal thickness of 0.2 µm). (**f**,**g**) EDS analysis of NiP deposit: the P content is in the order of 12.4 w%.

In order to assess the adherence, the soundness (absence of pores, cracks and breakages), the constituents of the different layers of the coating and their chemical composition, FIB-SEM examinations were performed on the coating of a PM adapter having followed the above temperature-humidity cycle. EDS mapping was also performed in order to identify the boundary between the substrate and the coatings and the nature of the different layers. A FIB cross-section was taken of the entire layer (Figure 12). The three layers of the coatings are identified, namely NiP (thickness between 10.7 µm and 13.2 µm) close to the substrate, electrolytic pure Ni (intermediate layer, 6.0 µm to 6.4 µm thick) and Au (top layer, 0.6 µm thick). The results show excellent wetting by the electroless NiP deposit of the C_f_s and the Al matrix, including in the narrow interface volumes between the matrix and the fibres. This result is as expected, taking into account that NiP coatings are generally highly uniform and not very sensitive to surface geometry (presence of recesses, sharp edges, etc.). The electrolytic Ni deposit also shows good wetting of the underlying NiP. The P content in the NiP layer could be estimated by EDS analysis in the order of 12.4 w% (Figure 12f,g). The coated parts, submitted to the above-mentioned temperature-humidity cycle, showed no deformation, proving that the coating is impervious to moisture. Consistently, examinations of the coated surfaces and cross-sections revealed no discontinuities or pinholes in the coating layer and no occurrence of corrosion phenomena under the coating respectively.

**Figure 13:**
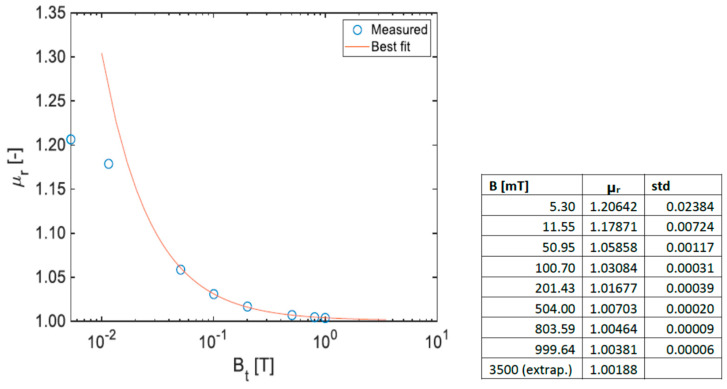
Magnetic permeability of the coated PM specimen as a function of the test field. Measurements at respectively 5 mT, 10 mT, 50 mT (using a Hall teslameter), 100 mT, 200 mT, 500 mT and 1 T (NMR teslameter) were performed. The saturation field of pure Ni is of the order of 0.64 T; however, since the permeability at the different fields is measured as secant from the origin, a continuous decrease is observed even above this field.

Magnetic permeability measurements were performed by Foerster Magnetoscop (under a field of 0.1 T) and SSM technique on a PM-coated adapter to assess the contribution of the coating to the magnetic permeability of the parts. For comparison, an uncoated adapter was also measured. The Foerster Magnetoscop results are *µ_r_* = 1.000 for the uncoated specimen and *µ_r_* = 1.013 for the coated specimen, respectively. The uncoated specimen mainly consists of aluminium and C_f_; ferromagnetic contributions to the relative permeability (equal to 1) are not detected. On the other hand, the relative permeability of the coated specimen is significantly higher due to the contribution of the ferromagnetic Ni coating. Indeed, electroless NiP can be obtained as non-magnetic for high P coatings (usually with over 11% P); nonetheless, the coating is slightly ferromagnetic, which is due to the presence of the electrolytic pure Ni layer of a thickness in the order of 6 µm between the NiP and the Au layers. Moreover, the values measured by the Foerster Magnetoscop may be underestimated because the specimen is not compliant with the instrument specifications in terms of thickness and flatness. Considering this, the coated specimen was selected for a further test campaign by SSM in order to get more accurate results at different field values (*B_t_*). Specimens were placed under uniform magnetic fields ranging between 0.005 T and 1.0 T (Figure 13). The results of magnetic permeability were then extrapolated to 3.5 T, which is the field at which the parts are submitted in operation. As the following law applies in the saturation region: *µ_r_ (H) =* 1 + *M_sat_/H*, where *H* is the magnetic field strength and *M_sat_* the magnetisation at saturation, the permeability at 3.5 T could be extrapolated using a first-order polynomial of 1/*H*, by fitting the data measured between 100 mT and 1 T. The value of *µ_r_* at 0.1 T is 1.03084, compared with the value issued from the Foerster Magnetoscop (*µ_r_* = 1.013). In addition to the geometry aspects mentioned above and to the fact that the SSM permeability is measured as secant from the origin, hence overestimated with respect to the tangent value for the same field, the difference can be explained since SSM integrates the effect of the coating on the whole surface of the adapter, while the Foerster Magnetoscop is only sensitive to a small volume of the specimen, i.e., to the local contribution of the coating from a single face. In any case, even SSM results show a value as low as *µ_r_* = 1.00381 already at 1 T, well below the 1.005 reference limit set by CERN for a material to be considered “non-magnetic”. The extrapolated *µ_r_* at 3.5 T is even lower (*µ_r_* = 1.00188).

## 4. Discussion

The observed degradation and dimensional changes of the powder metallurgy adapters submitted to severe temperature-humidity cycles are understood as due to an extreme moisture sensitivity induced by active galvanic corrosion phenomena between the C_f_s and aluminium-based matrix, inducing changes of volumes and even failures in the glued joint in the subassemblies due to the stresses introduced at the interfaces by the bending of the adapter (bi-material effect). The PM fabrication route minimises the number of cavities in the microstructure, mainly present at the Al/C_f_ interface, thanks to the applied semi-liquid phase sintering involving the presence of a small fraction of the liquid phase during the sintering process. Indeed, PM Al/C_f_ composite is a nonreactive system, where chemical reactions between the matrix, constituted by powder particles featuring an alumina surface layer, and the fibres are absent, while the semi-liquid phase sintering involving eutectic AlSi allows the wettability and the Al/C_f_ interfacial properties to be improved, particularly for high content of C_f_. On this basis, physical properties and, in particular, thermal conductivity compatible with requirements for heat sink materials could be obtained through this route [16]. Nevertheless, the present study has demonstrated that the internal structure of the investigated PM samples remains more open than for GPI samples and features an unfilled network of cavities between the C_f_s and the matrix, which may grow under the effect of the intake of moisture into the bulk material and subsequent galvanic corrosion. On this basis, subassemblies, including uncoated PM adapters, remain stable only if maintained within controlled laboratory conditions (22 °C and RH < 60%), where no dimensional effects have been measured for at least a time period of a few months. Since excursions to higher temperature and humidity levels cannot be excluded during the lifetime of HEP detectors before and after installation due to manufacturing, assembly, transport and maintenance periods where exposure conditions may be ill-controlled, PM parts have to be coated to make them suitable for such detectors.

The studied subassemblies, including coated PM adapters, have been shown to be dimensionally stable and microstructurally sound. PM adapters coated with a sequence of NiP, Ni and Au deposits are impervious to moisture. The coated PM parts object of the present study would be acceptable for TBPS ring production. Indeed, their relative magnetic permeability for fields above saturation of Ni (0.65 T), such as the operating field of the CMS magnet (3.5 T), is below 1.005, as shown in this study. However, the addition of a heavy-density coating approximately 20 µm thick induces an increase of the radiation length of the parts expected to be up to 15%; hence the thickness of the coatings should be carefully controlled and, as needed minimised for future productions.

On the other hand, products obtained by pressure infiltration casting (GPI) that have been the object of early development and prototyping for the main CERN LHC detectors, such as CMS and ATLAS, have shown less sensitivity even to extreme temperature-humidity cycles (50 °C, 100% RH for 144 h and 70 °C, 100% RH for 60 h). They may be used uncoated in the detector application presented here. Their compact microstructure explains the absence of observable galvanic corrosion phenomena on their surface and bulk, despite the observed presence of closed porosity and segregation band defects in their microstructure.

## 5. Conclusions and Perspectives

The present work confirms the suitability of the two investigated Al/C_f_ MMCs for application to the highly sensitive HEP detector components of the CERN CMS Outer Tracker, requiring tight geometrical control and microstructural stability over time. For powder metallurgy parts sintered through the semi-liquid phase process, it has been shown that a multi-layered protective noble metal coating is impervious to moisture, hence allowing dimensional stability to be guaranteed and the onset of corrosion phenomena to be avoided. The present investigations have also confirmed that the pressure infiltration casting Al/C_f_ product examined in the present work is already state-of-the-art for application to HEP detectors such as the CERN CMS Outer Tracker.

In order to circumvent the limitations of uncoated PM products highlighted in the present paper, alternative PM products obtained by an Al + Mg powder route may be considered and are being studied, with the aim of obtaining a denser microstructure and reducing porosity. An Al15%volMg/C_f_/55f product, hot pressed at 500 °C under 60 MPa, could achieve a hardness of 50 HV, equivalent to GPI MetGraf™ 4–230 samples, a compact structure at the Al alloy matrix-C_f_ interface and a density very close to the theoretical one [22]. It will be further studied. Alternative sintering processes based on Spark Plasma Sintering (SPS) have also been developed. In particular, parts based on SPS AlSiMg and Al + eutectic AlSi alloyed powders, respectively, have been produced and investigated. These products show moisture sensitivity and mechanical properties equivalent to or in excess of the ones of the GPI product examined in this study [23]. Optimization of the coatings applicable to PM products is also envisaged, taking into account that such coatings influence radiation length. It is envisaged to avoid the pure electrolytic Ni layer in order to further decrease magnetic permeability at low fields and to reduce the thickness of the NiP layer in order to decrease radiation length. On this basis, a non-magnetic coating exclusively based on NiP+Au deposits has been developed and will eventually be examined. Concerning the GPI product, future optimization will reduce the presence of segregation bands. It is known that these bands and the porosity embedded in them can occur even in casting with relatively thin cross-sections as the adapters [24]. Since they are generally due to excessive pressure during casting, closer control of the infiltration casting parameters should allow us to avoid them in series production.

## Data Availability

Not applicable.
